# Modelling of crowded polymers elucidate effects of double-strand breaks in topological domains of bacterial chromosomes

**DOI:** 10.1093/nar/gkt480

**Published:** 2013-06-05

**Authors:** Julien Dorier, Andrzej Stasiak

**Affiliations:** ^1^Center for Integrative Genomics, Faculty of Biology and Medicine, University of Lausanne, 1015-Lausanne, Switzerland and ^2^Vital-IT, Swiss Institute of Bioinformatics, 1015-Lausanne, Switzerland

## Abstract

Using numerical simulations of pairs of long polymeric chains confined in microscopic cylinders, we investigate consequences of double-strand DNA breaks occurring in independent topological domains, such as these constituting bacterial chromosomes. Our simulations show a transition between segregated and mixed state upon linearization of one of the modelled topological domains. Our results explain how chromosomal organization into topological domains can fulfil two opposite conditions: (i) effectively repulse various loops from each other thus promoting chromosome separation and (ii) permit local DNA intermingling when one or more loops are broken and need to be repaired in a process that requires homology search between broken ends and their homologous sequences in closely positioned sister chromatid.

## INTRODUCTION

A typical bacterium, such as *Escherichia coli*, stores its genetic information in just one several mega base pair-long circular DNA molecule that is ∼1000 times longer than the bacterium cell length (1,2). Such a long DNA molecule forms bacterial chromosome, whose structure is not yet determined in details, but several independent methods revealed that its organization is based on sequentially arranged, torsionally constrained loops, called topological domains (3). The size estimates of topological domains range from 10 000 to 700 000 bp (3–6). Because of steric and topological exclusion of neighbouring loops, the entire chromosome gains a character of a springy, but relatively rigid, circular filament (6–8) that is mechanically constrained within bacterial cell just like an elastic O-ring placed in a confining cylinder with a smaller diameter than the natural diameter of the O-ring. The topological exclusion naturally arises as a consequence of DNA decatenation activity of type II DNA topoisomerases that use the energy of adenosine triphosphate (ATP) hydrolysis to simplify DNA topology (9).

Mutual exclusion of relatively rigid sister chromosomes in the cylindrical confinement of bacterial cells causes that after DNA replication is finished, the two sister chromosomes settle one after the other along the long axis of rod-like bacterial cells (1,10–13). Such a placement of sister chromosomes prepares cells for the division, during which a forming septum constricts the cylindrical bacterial cell in the middle and splits the mother cell in such a way that each daughter cell receives one entire copy of the chromosome. Mutual exclusion between two sister chromosomes was proposed to be the main factors ensuring separation of bacterial chromosomes (14), but there are also known active mechanisms of DNA pumping, pushing and pulling that may assist that process (1,2,15–17).

Topological exclusion between different topological domains naturally decreases contacts between regions belonging to these topological domains. There are, though, specific biological situations where extensive contacts between DNA regions originally belonging to two different loops in two sister chromosomes should be allowed. Such a situation arises when there are one or more double-strand breaks in the DNA. These breaks need to be repaired by homologous recombination that necessities, though, that the DNA from the broken loop could invade the territory of unbroken loops in the search of homology. In that vital DNA repair pathway, the break is mended in a complex process that requires that at least one end of the broken region finds its homologous sequence in the sister chromosome (18–20). Although specialized proteins such as RecA (21) are needed for finding the homology, the search for homology is diffusion-driven, and RecA can only stabilize the homologous contacts that happened randomly (22–25). Therefore, the possibility to intermingle of linear DNA with closed topological domains is the prerequisite for the efficient homology recognition.

If there is a spontaneous switching between separated and mixed state upon DNA break this could resolve a conflict between the requirement for inter-domain topological exclusion as needed to give bacterial chromosomes the character of elastic O-rings and the requirement for inter-domain mixing as needed for double-strand break repair.

Many earlier studies using numerical simulation and theoretical approaches revealed that although highly concentrated linear polymers intermingle with each other, the non-catenated circular polymers stay segregated (26–29). However, these earlier studies treated mainly the case of a bulk solution, and it was realized relatively recently that the propensity of polymers to segregate increases when the polymers are confined to elongated microscopic vessels such as nanofabricated microchannels or cylindrical bacterial cells (14,30). One seminal study arrived to the conclusion that both circular and linear long polymeric chains are expected to segregate spontaneously when confined to microscopic cylindrical containers with the dimensions of *E. coli* cells (14). More recent simulation studies revealed, however, that two cylindrically confined linear polymers can mix with each other if their concentration is high enough and if these polymers are thin enough with respect to the diameter of the confining cylinder (31). That study establishing the influence of the polymer thickness on the mixability of linear polymers under cylindrical confinement did not address, though, the influence of thickness on the propensity to mix between circular polymers or between linear and circular polymers under a cylindrical confinement. As the cylindrical confinement can only aid the separation process (14,30,31); therefore, even thin circular polymers should not be expected to mix when confined within narrow cylinders. The remaining question then is whether linear and circular polymers can spontaneously mix under conditions of cylindrical confinement.

## MATERIALS AND METHODS

In each analysed case, simulated systems consisted of two chains (of same length) enclosed within a cylinder with spherical caps. Each chain, representing one topological domain, could be either circular or linear and was formed by N hard-core beads of radius *r*, with distance between centres of consecutive beads fixed to 2*r*, the N ranged from 200 to 1400.

To study thermal equilibrium properties of the system, we used a Metropolis Monte Carlo algorithm (32) based on crankshaft moves described earlier (33). The error bars were evaluated using the ‘blocking’ method (34). In the case of two circular chains, the chains were restricted to unknotted and uncatenated topology by preventing chain crossing during crankshaft moves (33). Presented data were obtained in simulations that started from an initial configuration with both chains inside the enclosing cylinder and not overlapping. We checked, though, that in case of two circular chains, these segregate even if we start with completely overlapping configurations (Supplementary Figures S6 and S7). The Metropolis Monte Carlo algorithm was run during >10^7^ Monte Carlo iterations (Supplementary Table S1) for each analysed system. We checked the decay of the correlation between measured variables during time evolution of studied systems and saw that in the worst case (two linear chains with 2800 beads), our simulations lasted for ∼1000 correlation times measured for the variable with the largest correlation time (Supplementary Table S1 and Supplementary Figures S1–S3). We also traced the temporal evolution of observable values (Supplementary Figures S4–S6). During the simulation, we measured contact probabilities as well as chain overlap length as defined later in the text.

The chain overlap length λ was defined as the length of the zone over which the two chains overlap along the axis of the confining cylinder (31).

To evaluate the average inter-chain contact probability experienced by individual beads, we considered all the possible pairs of beads with one bead in each chain and measured the ratio between the number of pairs showing a contact (two beads were considered in contact when the distance between their centres was smaller than 3*r*) and the total number of possible pairs. Similarly, average intra-chain contact probability was evaluated by considering all possible pairs of non-consecutive beads in the same chain and measuring the ratio between the number of pairs that were in contact and the total number of considered pairs.

To evaluate end-beads’ inter-chain contact probability, we considered all possible pairs of beads involving one of the end-beads of one chain and any of beads in the other chain and measured the ratio between the number of pairs showing the contact and the number of all considered pairs. To measure end-bead intra-chain contact probability, we considered all possible pairs involving an end-bead and any internal bead that is non-consecutive to that end-bead; subsequently, we measured the ratio between the number of the pairs with contact and the number of all considered pairs.

Thermal averages of contact probabilities and chain overlap length were obtained by measuring these quantities every 20*N* iterations (discarding the first 2000*N* iterations) and averaging. Error bars were evaluated with the ‘blocking’ method (34).

## RESULTS

To investigate whether independent topological domains can switch between segregated and mixed state upon DNA break, we proceeded with Monte Carlo simulations. As the precise organization of bacterial chromosomes is not known yet and we do not know, for example, how neighbouring or more distant loops are connected with each other, we investigated not only most elementary but also most generic cases of two circular, two linear or one circular and one linear long polymer molecules confined to a small cylindrical compartment with the geometry resembling this of *E. coli* cell. Such a system is certainly oversimplified, as the number of topological domains in bacterial cells with two sister chromosomes should be at least 10 times higher (4,6); however, the topological repulsion between ring polymers acts between each pair of interacting rings. To investigate the most generic case with the minimal number of adjustable parameters, we neglected DNA supercoiling. From a biological point of view, this neglection is at least partially justified when considering conditions under which environmentally induced double-stranded breaks are produced. Evolutionarily significant DNA damage sources, such as ultraviolet, produce many more single-stranded breaks than double-stranded breaks (35). Therefore, ultraviolet exposure that results in linearization of one or more topological domains is likely to torsionally relax many other topological domains. Our modelling neglects also specific interactions with nucleoid-associated proteins and possible osmotic compaction of bacterial nucleoids, which are likely to have profound effects on the overall structure of the nucleoid (36–38). However, as the knowledge about these interactions is far from complete, their effect is neglected also by other contemporary simulation studies investigating general underlying principles governing the behaviour of bacterial chromosomes (6,14,39).

Earlier studies revealed that the propensity of polymers to mix depends, in addition to the topological state of the polymers (linear or circular), on such factors like the concentration of the polymers, the aspect ratio of the confining cylinder and the ratio between the diameter of the confining cylinder and the confined polymer (31,40). To reflect the situation within *E. coli* cells, the total volume of modelled polymer chains was set to 5% of the actual volume of the confining cylinder. The 5% value was chosen, as it approximately corresponds to the volume occupied by two DNA molecules that are 2.2-mm long each, have the effective diameter of ∼3 nm (41) and are contained within a cell with a volume of ∼0.6 µm^3^ (42). The confining cylinder with hemispherical caps had a length:diameter ratio of 4, as this approximately corresponds to length:diameter ratio of typical *E. coli* cells (43). For our simulations of long polymeric chains, we used a model composed of freely jointed spherical beads (33). Such models are frequently used to model large-scale behaviour of polymers while not being too heavy computationally, and thus permits to perform computations in reasonable time (44). An earlier, widely quoted study arrived to the conclusion that under cylindrical confinement, similar to this operating in *E. coli* cells, concentrated polymer molecules segregate irrespectively of whether they are circular or linear (14). We believe, however, that this lack of distinction between mixability of linear and circular polymer molecules resulted from a too coarse graining of modelled polymers and in particular from the fact that the ratio between the diameter of the enclosing cylinder (D) and the diameter of enclosed polymers was too low (that ratio amounted to ∼14, whereas it is 100 times bigger in bacterial cells). To evaluate the influence of the ratio between the diameters of confining cylinder (D) and confined polymers (d), we performed a series of independent simulations, where the confining cylinder and the physical volume of modelled polymer chains were staying the same, but where the confined polymers were getting thinner as their length was increased. We studied equilibrium properties of systems composed of pairs of equal size chains amounting together to 400, 800, 1600 or 2800 beads. We investigated situations where both chains were circular, both were linear or where one was circular and the other linear. In the case of two circular chains, we introduced a condition that the circles remain unknotted and uncatenated, as it is known that bacterial topoisomerases such as topo IV use the energy of ATP hydrolysis to maintain the DNA in unknotted and uncatenated state (9,45).

**Figure 1.**
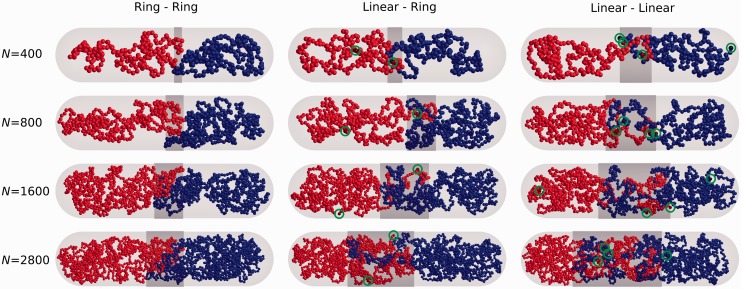
Presentation of simulated systems. Representative snapshots of equilibrated configurations of two circular (left column), circular and linear (middle column) and two linear chains (right column) with the increasing chain length and decreasing thickness so that they maintain the same volume. As representative, we consider those snapshots that show the overlap length λ close to the average value of λ for a corresponding system. The overlap regions are indicated with dark grey colour for an easy estimation of the overlap length λ. For easier visualization of end beads in linear chains, we encircled them on the shown images.

Figure 1 shows representative simulation snapshots of thermally equilibrated pairs of polymeric chains. The left column shows that as the two linear polymers get longer and thinner, they pass from a segregated to a mixed state. In the case of co-confined linear and circular chains (middle column), the onset of mixing is delayed as compared with two linear chains but chains with 1400 beads show a clear mixing. In the case of co-confined circular chains (right column), segregation is observed even for the thinnest chains tested.

As we progress from chains with 200 beads to these with 1400 beads, the ratio between the diameter of the enclosing cylinder and diameter of individual beads (D/d) increases, and in this case, it grows from ∼11 to ∼24. It is good to mention that in bacterial cells, the ratio between the inner diameter of the cell and the effective diameter of DNA is of the order of 150, however, reaching this D/d ratio in simulations that maintain the same concentration of modelled polymers would require us to increase the number of beads so much that we would be unable to sample well the configuration space of the system and provide a reliable statistics. Our results indicate, though, that for polymer concentration of ∼5%, the D/d ratio of ∼20 is sufficient to observe a pronounced mixing of linear and circular chains, and the extent of mixing is likely to increase with the further increase of the D/d ratio. In the case of two circular chains, we know from earlier theoretical and numerical studies that even at arbitrarily high D/d ratio (the situation in the bulk solution) and at high-polymer concentration, the polymer rings remain segregated (26–29,46). Therefore, there is no reason to suspect that a change of the D/d ratio from 22 to 150 could induce mixing of circular chains.

Translating the results obtained for the thinnest investigated chains to a biological situation, where there is a double-stranded break in one of topological domains of bacterial chromosome, that break naturally provides a possibility for the broken ends to invade territories occupied by other topological domains. This of course increases the probability that homologous contacts will be established between DNA sequences flanking the break and homologous sequences in the sister chromatid. The search is facilitated by the fact that two sister chromatids are maintained in a close apposition for a relatively long time after DNA replication (47,48). These homologous contacts are necessary for the two broken DNA ends to be reunited.

**Figure 2.**
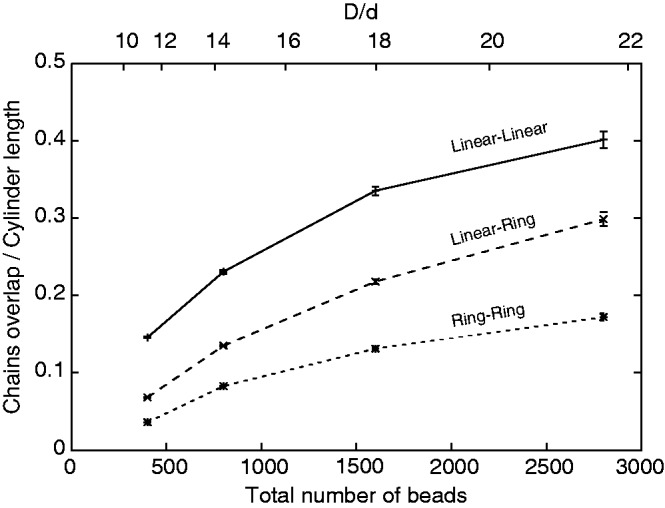
Chain overlap length λ increases with the chain length and the ratio between diameters of the confining cylinder and of confined chains. For all tested conditions, the overlap length was largest for systems composed of two linear chains and smallest for systems composed of two circular chains. The two circular chains showed also the smallest increase of the overlap length, as the chains were getting longer and thinner.

To provide a more quantitative measure of how a break of one or both chains stimulates mixing of two confined polymers, we followed the approach of Jung *et al.* (31) and measured the overlap length λ, i.e. the length of the zone over which the two chains overlap along the axis of the confining cylinder (Figure 1). The λ is negative when there is an empty zone between the two polymers, is zero if the two polymers just touch each other and can range up to the entire cylinder length when the polymers are mixed completely and the zone of overlap spans the entire cylinder length. Figure 2 shows how the λ/(cylinder length) changes as the chains become longer and thinner. It is visible that as the chains get longer and thinner and their D/d ratio increases from 11 to 22, the overlap length increases for all tried combinations. This increase is strongest for two linear chains but is also strong for linear and circular chains placed together. Two circular chains also show an increase of the overlap length, but this increase is much less pronounced and seems to saturate.

**Figure 3.**
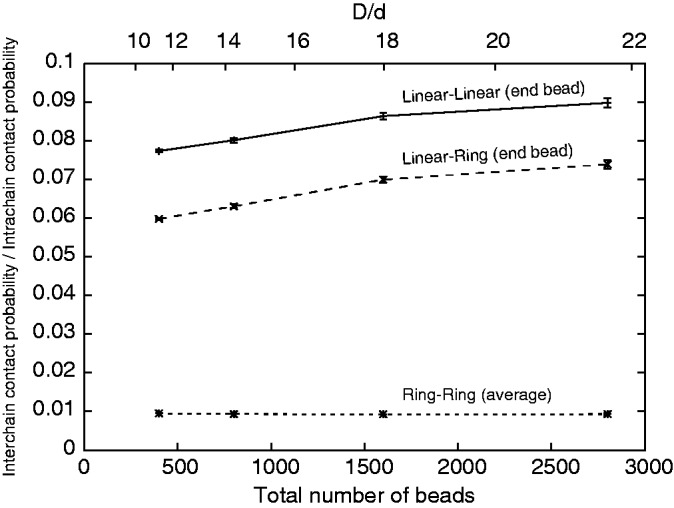
End-beads in linear chains show much higher inter-/intra-chain contact ratio than the corresponding ratio measured for an average bead in systems composed of two circular chains.

Of course, chains with a larger overlap length λ are more likely to contact each other than chains with a smaller overlap length; however, the overlap length by itself does not translate directly into the probabilities of contacts involving the two chains. Therefore, we investigated how the ratio between inter- and intra-chain contacts is affected by the topology of two polymer chains subject to cylindrical confinement, as the chains are getting longer and thinner while maintaining the same overall volume fraction (Figure 3). In the case of two circular chains, we looked at the ratio between inter- and intra-chain contacts experienced by an average bead in the chain. The overall connectivity of the chain makes intra-chain contacts much more frequent than inter-chain contacts. The lowest profile in Figure 3 shows that any bead in a circular chain is >100 times more likely to contact another bead from the same chain than a bead from the second chain. This high dominance of intra-chain contacts remains practically unchanged as the circular chains get longer and thinner.

Snapshots presented in Figure 1 reveal that mixing between linear and circular chains is frequently limited to terminal portions of linear chains. In fact for homologous pairing of broken ends with the sister chromosome, it is not required that the entire linear DNA molecule mixes with the circular DNA molecule. The only requirement is that two or at least one end of the broken molecule can mix with the other molecule so that the homology can be found. Therefore, we investigated the relative frequency of inter-chain and intra-chain contacts experienced by terminal beads of linear chains. We observed that inter-chain/intra-chain contact ratio of terminal beads in linear chains is several fold higher than inter-chain/intra-chain contact ratio of an average bead in circular chain confined together with another circular chain. In the case of systems composed of one linear and one circular chain (Figure 3, middle profile) that relative increase of intermolecular contact of terminal beads was ∼6-fold for chains composed of 200 beads and ∼8-fold for chains composed of 1400 beads. In the case of systems composed of two linear chains (Figure 3, lower profile), the boost of inter-chain/intra-chain contacts experienced by terminal beads was even stronger and neared an ∼10-fold increase for chains of 1400 beads as compared with the inter-chain/intra-chain contact ratio experienced by any bead of circular chains of the same size.

## DISCUSSION

Our modelling studies indicate that a double-strand break in a closed topological domain within bacterial chromosome is able to induce a spontaneous transition between a segregated and intermingled state involving topological domains belonging to two sister chromatids kept in close apposition (47).

**Figure 4.**
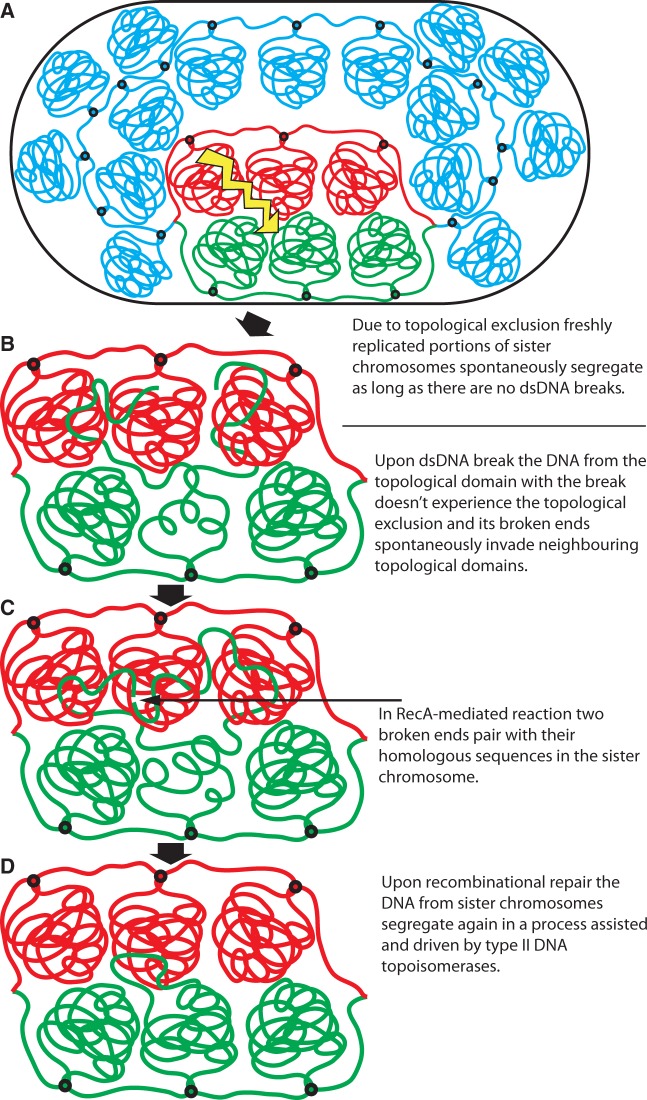
DNA break-induced transition between DNA segregation and intermingling plays an essential role in the process of homology-dependent repair of the break. (**A**) Newly replicated portions of sister chromosomes because of a topological exclusion spontaneously segregate from each other and take positions preparing them for the ensuing cell division. (**B**) After dsDNA break, the linear portions flanking the break are free to invade other topological domains including the one with the homologous sequences in the sister chromosome. (**C**) RecA-mediated homologous pairing leads to rejoining of the two broken ends. (**D**) After circular nature of the DNA is re-established, the action of topoisomerase IV helps to remove possible interlinks between re-established topological domain and other topological domains.

Figure 4 schematically presents a model showing how a transition between segregated and intermingled state of topological domains in freshly replicated portions of bacterial chromosomes can be implicated in dsDNA break repair.

Figure 4A illustrates that as long as there is no DNA damage resulting in a dsDNA break, freshly replicated portions of sister chromosomes segregate from each other, as even at high concentration of polymers, closed polymeric loops effectively repulse each other especially when they are actively maintained in uncatenated state (28,29,46). Of course, to reach non-catenated segregated state of topological domains belonging to freshly replicated portions of sister chromosomes, the action of DNA topoisomerases is needed. In this respect, especially important is the activity of bacterial topoisomerase IV and DNA gyrase that are required for active DNA decatenation (9,41,49,50).

Once a dsDNA break is formed in one of the topological domains, the DNA portions with free ends are released from the topological exclusion. For these linearized portions of topological domains, it becomes entropically favourable to intermingle with neighbouring topological domains including this formed on the homologous portion of the sister chromosome (Figure 4B and C).

Homology-dependent repair of dsDNA break requires pairing between sequences flanking the break and their homologous sequences that for any single-copy gene can only be found in the sister chromosome. The actual pairing process necessitates participation of specialized proteins such as RecA that modify the structure of the broken ends in such a way that they can form stabilizing hydrogen bonds when contacting homologous DNA sequence (51,52). However, the actual search process is diffusion-driven (22–25), and homologous contacts are only established when homologous regions find each other after a random search. Once the pairing is achieved, the two ends can be joined by several different mechanisms (19). If both ends paired with their homologous sequences, the ends are joined by formation and processing of double Holliday junction (53). If only one end managed to find its homologous sequence, it can follow synthesis-dependent strand annealing (SDSA) repair pathway, where the 3′-ended strand of the paired end is extended by DNA polymerase using the homologous sequence as the template (20). Subsequently, the extended end can be released from the homologous contact and anneal with the appropriately processed other end resulting from the initial double-strand break (20). That later process is facilitated by the fact that linearized polymers mix preferentially with each other (Figure 1).

When the continuity of the broken loop is re-established, the action of DNA topoisomerase IV permits removal of interlinks involving newly closed topological domain and other domains (Figure 4D). From that point on, both portion of freshly replicated sister chromosomes regain the character of an elastic O-ring because of strong topological repulsion acting along individual portions of sister chromosomes and also between the two sister chromosomes. These repulsive elastic interactions can drive then the process of separation of two sister chromosomes.

We believe that organization of bacterial chromosomes into topological domains was selected during the evolution, as it permitted to use generic polymer effects that not only cause spontaneous segregation of highly concentrated non-catenated polymeric rings but also cause spontaneous mixing of linear and circular polymers. Thanks to topological repulsion of sequential topological domains, bacterial chromosomes acquire the overall form of an elastic O-ring that is needed to ensure rapid segregation of newly replicated bacterial chromosomes (7,8). Topological repulsion ceases to act, though, when the DNA is linearized, and this provides the possibility of broken DNA to mix with juxtaposed topological domains on sister chromatid, as needed to establish homologous contacts required for repair of double-strand breaks.

Recent studies firmly established that also eukaryotic chromosomes are composed of topological domains (54,55). Several earlier simulation studies proposed that organization of eukaryotic chromosomes into loops promotes segregation of interphase chromosomes into distinct chromosome territories (56–59). Of course double-stranded breaks occurring in eukaryotic chromosomes are also expected to promote intermixing that would be required for the homology search needed for the repair of double-stranded breaks. Dedicated study aimed to observe consequences of double-stranded breaks in eukaryotic nuclei showed that specifically labelled chromatin regions surrounding double-stranded breaks expand by 30–40% within 180 s after the emergence of the breaks (60). These results strongly support the proposal that double-stranded breaks in topological domains induce a transition from segregated to intermingled state involving the topological domain in which the double-stranded break occurred.

## SUPPLEMENTARY DATA

Supplementary Data are available at NAR Online: Supplementary Table 1, Supplementary Figures 1–7 and Supplementary Methods.

## FUNDING

Swiss National Science Foundation [31003A_138367 to A.S.]. Funding for open access charge: The open access publication charge for this paper has been waived by Oxford University Press - NAR Editorial Board members are entitled to one free paper per year in recognition of their work on behalf of the journal.

*Conflict of interest statement*. None declared.

## Supplementary Material

Supplementary Data

## References

[1] Reyes-Lamothe R, Wang X, Sherratt D (2008). *Escherichia coli* and its chromosome. Trends Microbiol..

[2] Toro E, Shapiro L (2010). Bacterial chromosome organization and segregation. Cold Spring Harb. Perspect. Biol..

[3] Postow L, Hardy CD, Arsuaga J, Cozzarelli NR (2004). Topological domain structure of the *Escherichia coli* chromosome. Genes Dev..

[4] Worcel A, Burgi E (1972). On the structure of the folded chromosome of *Escherichia coli*. J. Mol. Biol..

[5] Higgins NP, Yang X, Fu Q, Roth JR (1996). Surveying a supercoil domain by using the gamma delta resolution system in Salmonella typhimurium. J. Bacteriol..

[6] Fritsche M, Li S, Heermann DW, Wiggins PA (2012). A model for *Escherichia coli* chromosome packaging supports transcription factor-induced DNA domain formation. Nucleic Acids Res..

[7] Wiggins PA, Cheveralls KC, Martin JS, Lintner R, Kondev J (2010). Strong intranucleoid interactions organize the *Escherichia coli* chromosome into a nucleoid filament. Proc. Natl Acad. Sci. USA.

[8] Chaudhuri D, Mulder BM (2012). Spontaneous helicity of a polymer with side loops confined to a cylinder. Phys. Rev. Lett..

[9] Rybenkov VV, Ullsperger C, Vologodskii AV, Cozzarelli NR (1997). Simplification of DNA topology below equilibrium values by type II topoisomerases. Science.

[10] Bates D, Kleckner N (2005). Chromosome and replisome dynamics in *E. coli*: loss of sister cohesion triggers global chromosome movement and mediates chromosome segregation. Cell.

[11] Wang X, Sherratt DJ (2010). Independent segregation of the two arms of the *Escherichia coli* ori region requires neither RNA synthesis nor MreB dynamics. J. Bacteriol..

[12] Joshi MC, Bourniquel A, Fisher J, Ho BT, Magnan D, Kleckner N, Bates D (2011). *Escherichia coli* sister chromosome separation includes an abrupt global transition with concomitant release of late-splitting intersister snaps. Proc. Natl Acad. Sci. USA.

[13] Wang X, Lesterlin C, Reyes-Lamothe R, Ball G, Sherratt DJ (2011). Replication and segregation of an *Escherichia coli* chromosome with two replication origins. Proc. Natl Acad. Sci. USA.

[14] Jun S, Mulder B (2006). Entropy-driven spatial organization of highly confined polymers: lessons for the bacterial chromosome. Proc. Natl Acad. Sci. USA.

[15] Aussel L, Barre FX, Aroyo M, Stasiak A, Stasiak AZ, Sherratt D (2002). FtsK Is a DNA motor protein that activates chromosome dimer resolution by switching the catalytic state of the XerC and XerD recombinases. Cell.

[16] White MA, Eykelenboom JK, Lopez-Vernaza MA, Wilson E, Leach DR (2008). Non-random segregation of sister chromosomes in *Escherichia coli*. Nature.

[17] Hui MP, Galkin VE, Yu X, Stasiak AZ, Stasiak A, Waldor MK, Egelman EH (2010). ParA2, a Vibrio cholerae chromosome partitioning protein, forms left-handed helical filaments on DNA. Proc. Natl Acad. Sci. USA.

[18] Kuzminov A (1999). Recombinational repair of DNA damage in *Escherichia coli* and bacteriophage lambda. Microbiol. Mol. Biol. Rev..

[19] Ayora S, Carrasco B, Cardenas PP, Cesar CE, Canas C, Yadav T, Marchisone C, Alonso JC (2011). Double-strand break repair in bacteria: a view from Bacillus subtilis. FEMS Microbiol. Rev..

[20] Gumbiner-Russo LM, Rosenberg SM (2007). Physical analyses of *E. coli* heteroduplex recombination products *in vivo*: on the prevalence of 5′ and 3' patches. PLoS One.

[21] Stasiak A, Egelman EH (1994). Structure and function of RecA-DNA complexes. Experientia.

[22] Honigberg SM, Gonda DK, Flory J, Radding CM (1985). The pairing activity of stable nucleoprotein filaments made from recA protein, single-stranded DNA, and adenosine 5'-(gamma-thio)triphosphate. J. Biol. Chem..

[23] Menetski JP, Bear DG, Kowalczykowski SC (1990). Stable DNA heteroduplex formation catalyzed by the *Escherichia coli* RecA protein in the absence of ATP hydrolysis. Proc. Natl Acad. Sci. USA.

[24] Rosselli W, Stasiak A (1990). Energetics of RecA-mediated recombination reactions. Without ATP hydrolysis RecA can mediate polar strand exchange but is unable to recycle. J. Mol. Biol..

[25] Forget AL, Kowalczykowski SC (2012). Single-molecule imaging of DNA pairing by RecA reveals a three-dimensional homology search. Nature.

[26] Cates ME, Deutsch JM (1986). Conjectures on the statistics of ring polymers. J. Physique.

[27] Müller M, Wittmer JP, Cates ME (1996). Topological effects in ring polymers: a computer simulation study. Phys. Rev. E Stat. Phys. Plasmas Fluids Relat. Interdiscip Topics.

[28] Suzuki J, Takano A, Matsushita Y (2008). Topological effect in ring polymers investigated with Monte Carlo simulation. J. Chem. Phys..

[29] Suzuki J, Takano A, Deguchi T, Matsushita Y (2009). Dimension of ring polymers in bulk studied by Monte-Carlo simulation and self-consistent theory. J. Chem. Phys..

[30] Teraoka I, Wang YM (2004). Computer simulation studies on overlapping polymer chains confined in narrow channels. Polymer.

[31] Jung Y, Kim J, Jun S, Ha BY (2012). Intrachain Ordering and Segregation of Polymers under Confinement. Macromolecules.

[32] Metropolis N, Rosenbluth AW, Rosenbluth MN, Teller AH, Teller E (1953). Equations of state calculation by fast computing machines. J. Chem. Phys..

[33] Dorier J, Stasiak A (2010). The role of transcription factories-mediated interchromosomal contacts in the organization of nuclear architecture. Nucleic Acids Res..

[34] Flyvbjerg H, Petersen HG (1989). Error estimates on averages of correlated data. J. Chem. Phys..

[35] Bonura T, Smith KC (1975). Enzymatic production of deoxyribonucleic acid double-strand breaks after ultraviolet irradiation of *Escherichia coli* K-12. J. Bacteriol..

[36] Odijk T (1998). Osmotic compaction of supercoiled DNA into a bacterial nucleoid. Biophys. Chem..

[37] Woldringh CL, Nanninga N (2006). Structural and physical aspects of bacterial chromosome segregation. J. Struct. Biol..

[38] Fisher JK, Bourniquel A, Witz G, Weiner B, Prentiss M, Kleckner N (2013). Four-dimensional imaging of *E. coli* nucleoid organization and dynamics in living cells. Cell.

[39] Jun S, Wright A (2010). Entropy as the driver of chromosome segregation. Nat. Rev. Microbiol..

[40] Jung Y, Ha BY (2010). Overlapping two self-avoiding polymers in a closed cylindrical pore: Implications for chromosome segregation in a bacterial cell. Phys. Rev. E Stat. Phys. Plasmas Fluids Relat. Interdiscip Topics.

[41] Martinez-Robles ML, Witz G, Hernandez P, Schvartzman JB, Stasiak A, Krimer DB (2009). Interplay of DNA supercoiling and catenation during the segregation of sister duplexes. Nucleic Acids Res..

[42] Kubitschek HE (1990). Cell volume increase in *Escherichia coli* after shifts to richer media. J. Bacteriol..

[43] Miao J, Hodgson KO, Ishikawa T, Larabell CA, LeGros MA, Nishino Y (2003). Imaging whole *Escherichia coli* bacteria by using single-particle x-ray diffraction. Proc. Natl Acad. Sci. USA.

[44] Hyeon C, Thirumalai D (2011). Capturing the essence of folding and functions of biomolecules using coarse-grained models. Nat. Commun..

[45] Liu Z, Zechiedrich L, Chan HS (2010). Action at hooked or twisted-hooked DNA juxtapositions rationalizes unlinking preference of type-2 topoisomerases. J. Mol. Biol..

[46] Vettorel T, Grosberg AY, Kremer K (2009). Statistics of polymer rings in the melt: A numerical simulation study. Phys. Biol..

[47] Weiner A, Zauberman N, Minsky A (2009). Recombinational DNA repair in a cellular context: a search for the homology search. Nat. Rev. Microbiol..

[48] Lesterlin C, Gigant E, Boccard F, Espeli O (2012). Sister chromatid interactions in bacteria revealed by a site-specific recombination assay. EMBO J..

[49] Zechiedrich EL, Cozzarelli NR (1995). Roles of topoisomerase IV and DNA gyrase in DNA unlinking during replication in *Escherichia coli*. Genes Dev..

[50] Witz G, Stasiak A (2010). DNA supercoiling and its role in DNA decatenation and unknotting. Nucleic Acids Res..

[51] Howard-Flanders P, West SC, Stasiak A (1984). Role of RecA protein spiral filaments in genetic recombination. Nature.

[52] Stasiak A (1992). Three-stranded DNA structure; is this the secret of DNA homologous recognition?. Mol. Microbiol..

[53] West SC (2009). The search for a human Holliday junction resolvase. Biochem. Soc. Trans..

[54] Dixon JR, Selvaraj S, Yue F, Kim A, Li Y, Shen Y, Hu M, Liu JS, Ren B (2012). Topological domains in mammalian genomes identified by analysis of chromatin interactions. Nature.

[55] Nora EP, Lajoie BR, Schulz EG, Giorgetti L, Okamoto I, Servant N, Piolot T, van Berkum NL, Meisig J, Sedat J (2012). Spatial partitioning of the regulatory landscape of the X-inactivation centre. Nature.

[56] Cook PR, Marenduzzo D (2009). Entropic organization of interphase chromosomes. J. Cell Biol..

[57] de Nooijer S, Wellink J, Mulder B, Bisseling T (2009). Non-specific interactions are sufficient to explain the position of heterochromatic chromocenters and nucleoli in interphase nuclei. Nucleic Acids Res..

[58] Marenduzzo D, Orlandini E (2009). Topological and entropic repulsion in biopolymers. J. Stat. Mech. Theory Exp..

[59] Dorier J, Stasiak A (2009). Topological origins of chromosomal territories. Nucleic Acids Res..

[60] Kruhlak MJ, Celeste A, Dellaire G, Fernandez-Capetillo O, Muller WG, McNally JG, Bazett-Jones DP, Nussenzweig A (2006). Changes in chromatin structure and mobility in living cells at sites of DNA double-strand breaks. J. Cell Biol..

