# Supplementary material for: Modelling of crowded polymers elucidate effects of double-strand breaks in topological domains of bacterial chromosomes
Source: Nucleic Acids Res. 2013 Jun 5;41(14):6808–15. doi: 10.1093/nar/gkt480 (PMC3737558; doi:10.1093/nar/gkt480)
Supplement: Supplementary Data [file supp_41_14_6808__index.html]

Modelling of crowded polymers elucidate effects of double-strand breaks in topological domains of bacterial chromosomes — Modelling of crowded polymers elucidate effects of double-strand breaks in topological domains of bacterial chromosomes — Supplementary Data 

# Modelling of crowded polymers elucidate effects of double-strand breaks in topological domains of bacterial chromosomes

## Supplementary Data

files

**Files in this Data Supplement:**

- Supplementary Data - pdf file
