# Supplementary material for: Modelling of crowded polymers elucidate effects of double-strand breaks in topological domains of bacterial chromosomes
Source: Nucleic Acids Res. 2013 Jun 5;41(14):6808–15. doi: 10.1093/nar/gkt480 (PMC3737558; doi:10.1093/nar/gkt480)
Supplement: Supplementary Data [file supp_gkt480_nar-00224-n-2013-File006.pdf]

Convergence of the Metropolis Monte Carlo algorithm

During the Monte Carlo simulation, we measured the value of each observable ("Chains overlap", "Interchain contact probability" and "Intrachain contact probability") every 10 Monte Carlo iterations. Let us denote by $\{A_1, A_2, \cdots, A_M\}$ the set of measurements for the observable A. For each observable A, in addition to the average value

$$\langle A \rangle = \frac{1}{M} \sum_{i=1}^{M} A_i.$$

we evaluated the error bars for $\langle A \rangle$ with the "blocking" method described by Flyvbjerg and Petersen (*1*), and also the autocorrelation $C_A(n)$

$$C_A(n) = \frac{\langle A_{i+n} A_i \rangle - \langle A_{i+n} \rangle \langle A_i \rangle}{\langle A^2 \rangle - \langle A \rangle^2}$$

Figures S1, S2 and S3 show the autocorrelation of the observables "Chains overlap", "Interchain contact probability" and "Intrachain contact probability" for the three models considered in this study with 2800 beads (largest systems). Correlation times, estimated by fitting $A \exp(-n/\tau)$ to autocorrelation curves, are given in table S1, along with the number of Monte Carlo iterations (1 Monte Carlo iteration=N crankshaft moves) for each model. In the worst case (Linear-Linear with N=2800), the number of Monte Carlo iterations corresponds to approximately 1000 largest correlation time. This result suggests that enough independant configuration were taken into account in the statistics.
Additionally, Figures S4, S5 and S6 present the evolution of the "Chains overlap" observable (which is among the slowest observables) as a function of the number of Monte Carlo iterations. These figures confirm that the system is well equilibrated.
Finally, for the model with two circular chains and 2800 beads, we also checked that even when starting from an initial configuration with overlapping chains (Figure S7), the two chains quickly segregate ($\sim 3 \cdot 10^5$ iterations, see Figure S8).

References

1. Flyvbjerg, H. & Petersen, H. G. Error estimates on averages of correlated data. J. Chem. Phys. **91**, 461-466 (1989).

Model	N	M	$\tau_{\text{Chains overlap}}$	$\tau_{P_{\text{inter}}}$	$\tau_{P_{\text{intra}}}$	$\frac{M}{\max_A(\tau_A)}$
Linear-Linear	400	$6.9 \cdot 10^7$	998	650	14	$6.9 \cdot 10^4$
Linear-Linear	800	$2.4 \cdot 10^7$	2346	1031	13	$1.0 \cdot 10^4$
Linear-Linear	1600	$2.3 \cdot 10^7$	5737	1656	13	$4.1 \cdot 10^3$
Linear-Linear	2800	$1.1 \cdot 10^7$	11700	2726	14	$9.7 \cdot 10^2$
Linear-Ring	400	$6.6 \cdot 10^7$	342	402	13	$1.6 \cdot 10^5$
Linear-Ring	800	$2.3 \cdot 10^7$	1029	648	13	$2.3 \cdot 10^4$
Linear-Ring	1600	$2.3 \cdot 10^7$	3794	1336	14	$6.1 \cdot 10^3$
Linear-Ring	2800	$1.1 \cdot 10^7$	8749	2460	15	$1.3 \cdot 10^3$
Ring-Ring	400	$2.4 \cdot 10^8$	116	382	11	$6.3 \cdot 10^5$
Ring-Ring	800	$9.2 \cdot 10^7$	502	468	187	$1.8 \cdot 10^5$
Ring-Ring	1600	$8.7 \cdot 10^7$	2902	3016	364	$2.9 \cdot 10^4$
Ring-Ring	2800	$3.8 \cdot 10^7$	17648	17833	850	$2.1 \cdot 10^3$

Table S1: Number of iterations M, correlation times τ_A and smaller ratio (Number of iterations)/τ_A for all models, number of beads N and observables A considered in this study. P_{inter} and P_{intra} denote respectively interchain and intrachain contact probability. Correlation time τ_A for observable A was obtained by fitting $B\exp(-n/\tau_A)$ to observable A autocorrelation curve $C_A(n)$ (see Figures S1, S2 and S3 for the case N=2800).

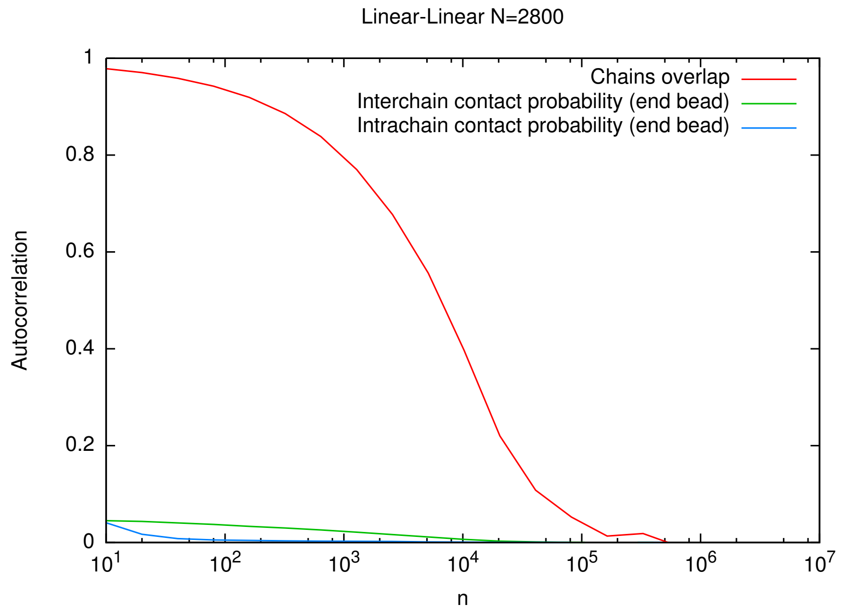

Figure S1: Autocorrelations of the measured observables for the model with two Linear chains and N=2800 beads.

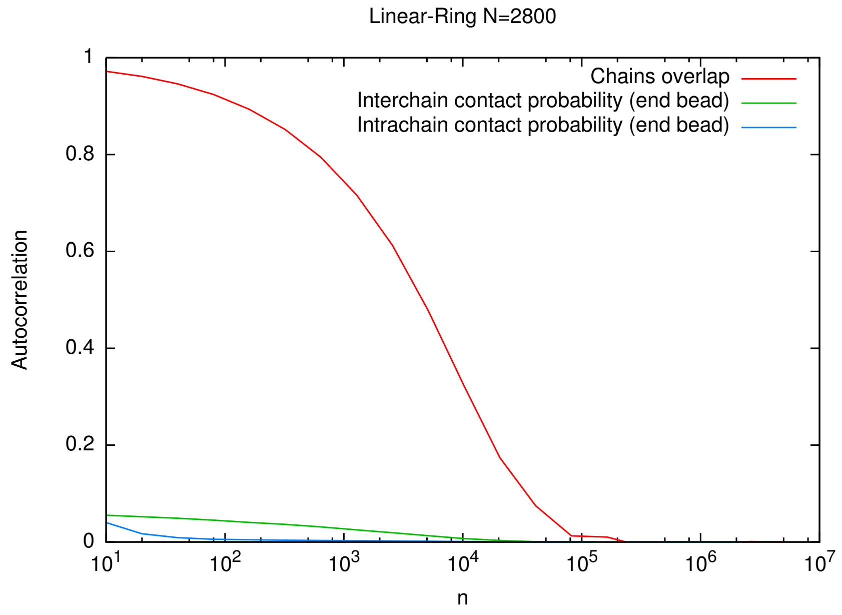

Figure S2: Autocorrelations of the measured observables for the model with one Linear chain and one circular chain and N=2800 beads.

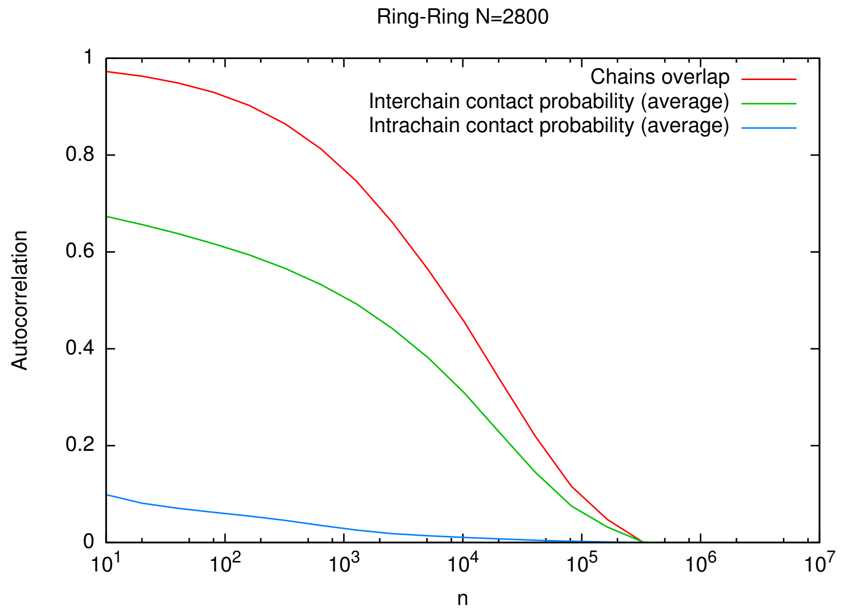

Figure S3: Autocorrelations of the measured observables for the model with two circular chains and N=2800 beads.

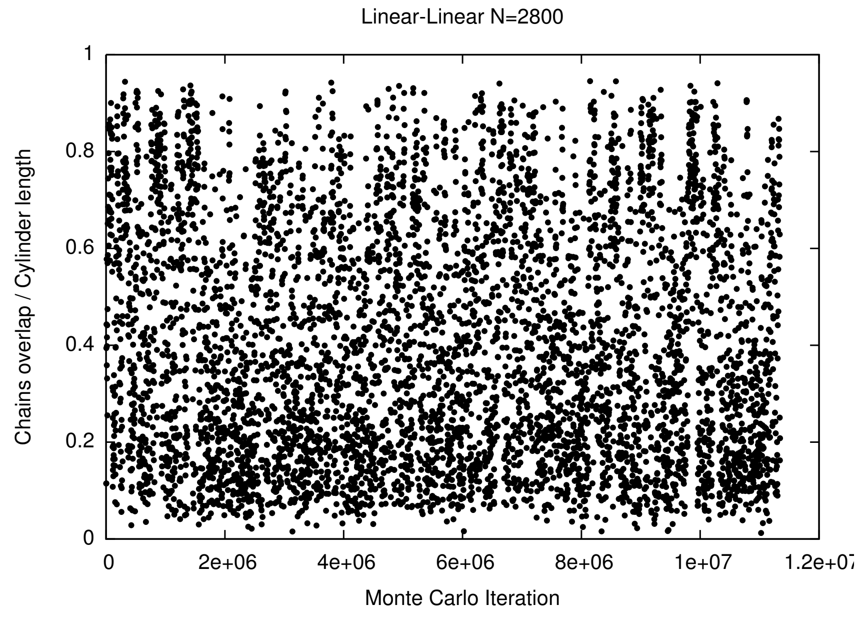

Figure S4: Evolution of chains overlap for the model with two linear chains and N=2800 beads.

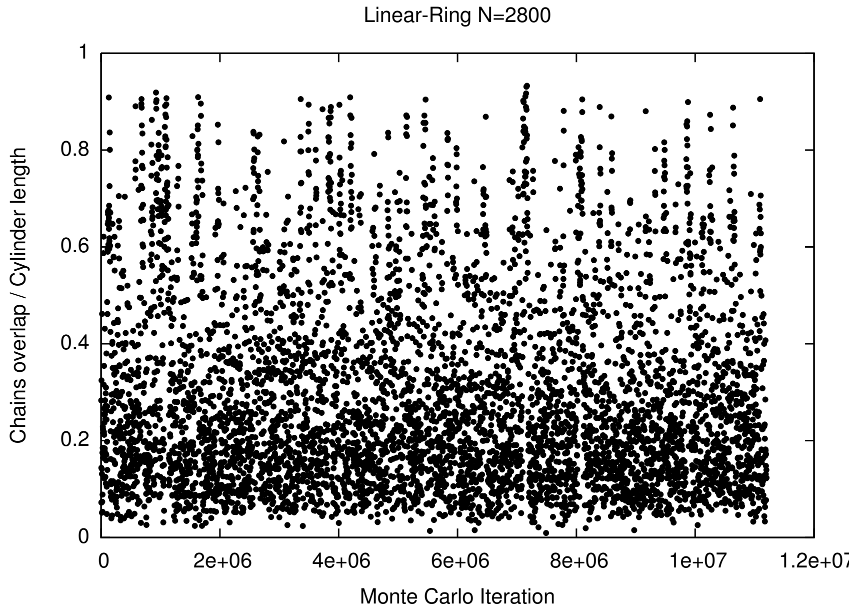

Figure S5: Evolution of chains overlap for the model with one linear chain and one circular chain and N=2800 beads.

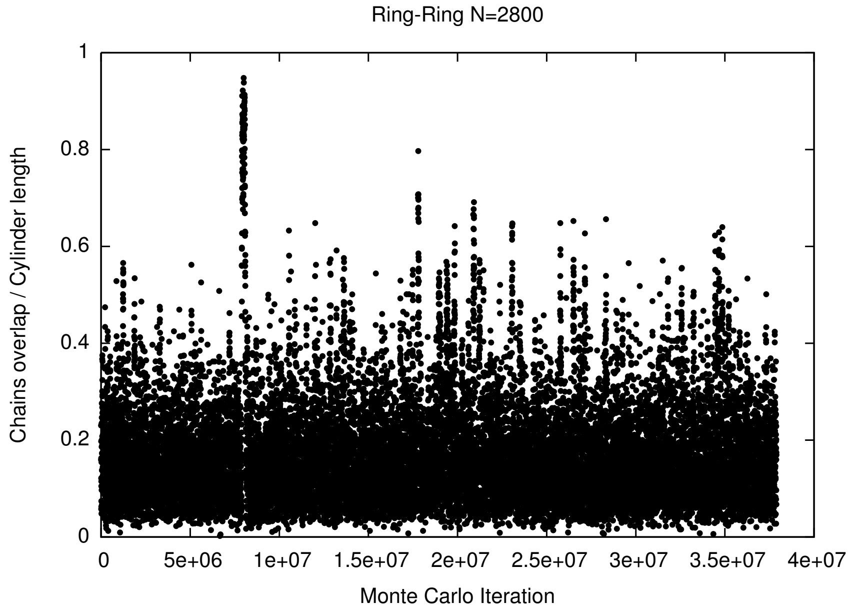

Figure S6: Evolution of chains overlap for the model with two circular chains and N=2800 beads.

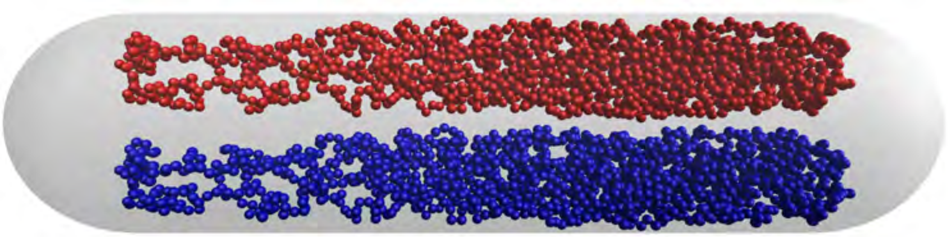

Figure S7: Initial configuration with overlapped chains (N=2800 beads).

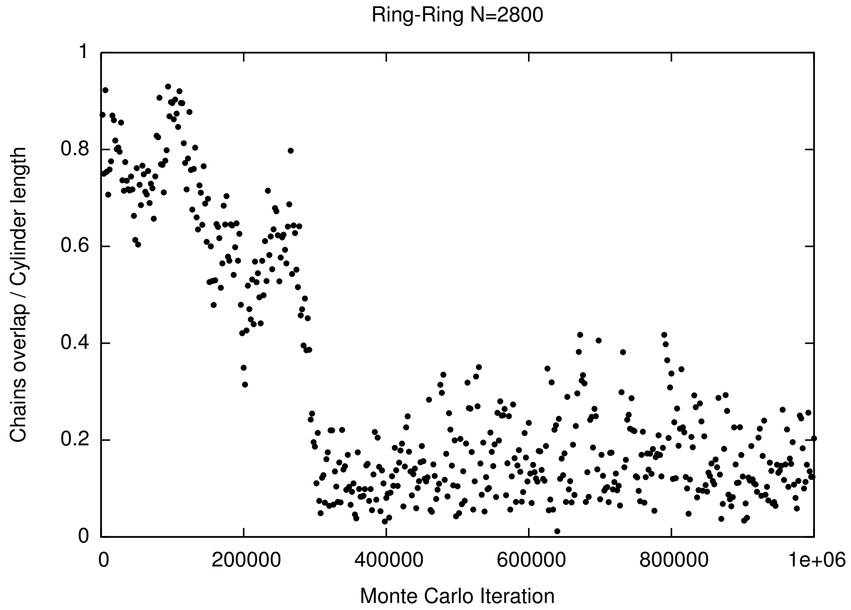

Figure S8: Evolution of chains overlap for the model with two circular chains and N=2800 beads, starting from an initial configuration with overlapped chains (Figure S7).
